# Medical News and Miscellany

**Published:** 1896-01

**Authors:** 


### Medical News and Miscellany.

**Happy New Year!**—The JOURNAL makes its best bow to its hosts of readers; begs to assure them of its keenest appreciation of their devotion through these trying years, and thanks them for their unwavering support, moral and financial. We wish them, one and all, a happy New Year!

**Bacteriological Report.**—We have received a copy of the report made to the Hartford Board of Health, by Dr. Arthur J. Wolff, their bacteriologist, in which he describes the mode and means of examining for the true diphtheria bacillus. He says: "These researches have proved beyond any doubt that any examination of the throat of a person suffering with inflammation with membraneous deposit or exudate leads to no correct diagnosis and is absolutely unreliable without a thorough bacteriological study; because a throat containing a small amount of membrane, or even none at all, may be exceedingly virulent with Klebbs-Lœffler bacillus, while, on the other hand, pseudo-diphtheria may be accompanied with a most extensive, thick and foul-smelling exudate; thus, the culture-tube and the microscope are the only means by which the difference can be determined." The doctor describes the process in detail. It is an interesting report, and a copy will be sent free by Dr. Wolff upon application. Dr. Wolff is the son of State Quarantaine Officer Wolff, at Brownsville, and was the expert bacteriologist in the famous Dr. Buchanan poisoning case.

---

**Dr. C. W. Simpson,** of Waxahachie, has been appointed county physician of Ellis connty.

---

**Dr. D. Berry,** of San Antonio, we see by the daily papers, has "been elected county physician of Bexar county by the county commissioners." Under the amended law, the county commissioners have nothing whatever to do with the choice or appointment of connty physician. The appointment rests solely with the connty judge. See section 14.

---

**Treatment of Chronic Constipation by Large Enemata of Oil.**—Dr. Carl Berger has obtained most successful results in obstipation from the "oil-cure" described by Fleiner, of Heidelberg, in 1893. Enemata of 400 to 500 cc. of good olive or poppy or sesame oil are used. The oil should be at body temperature and given slowly with moderate pressure. Fleiner recommended the dorsal position with elevated pelvis, but Berger now prefers the knee-elbow position. The operation takes twenty to thirty minutes. Then the patient lies, with hips raised, fifteen minutes on the left side and the same time on the right side. All patients feel the pressure of the oil in the transverse colon. Pain was present in only two out of forty-one cases, and twice the taste of oil was reported several hours after the enema. If the patient remains recumbent, a stool usually appears four or five hours later, but the real effect comes the following day in two or three spontaneous evacuations, often containing bile. Oil appears in the stools for five or six, or even ten days. Usually a spontaneous evacuation follows for a number of days, aided, no doubt, by proper care in choice of food, etc. Before further use of the oil, Berger waits until a necessity arises, when the enema is repeated. He finds that the intervals grow longer and that the ultimate success in even severe cases is greater than he has obtained by any other means. Rosenheim has published his experience and while less sanguine, recommends the procedure in connection with massage and electricity. Blum has noted good results in colic accompanying gall stones. The ease with which good results were obtained in hysterical and neurasthenic women makes Berger warm in his praise of this method, in comparison with massage of the abdomen, hydrotherapy or faradization of abdominal wall.—*Deutsche Medicin. Wochenschrift*, July 25, 1895.

---

The JOURNAL continues to prosper, thanks to its patrons. Its influence and usefulness, like the wings of "ye great American Eagle-bird," are spread *away out yonder!*

---

**The Transactions, Texas State Medical Association, 1895.**—The JOURNAL is in receipt of the annual volume of Transactions of the Texas State Medical Association for 1895, just issued by Secretary West. It is a little late, owing, we understand, to delay on the part of authors of papers in returning proof-sheets. The volume is much smaller than usual, containing only 277 pages. It is bound, uniform with former editions and keeps up the reputation of the secretary for taste and attention to business.

---

**Dr. M. K. Lott,** long time State quarantine officer at Eagle Pass, has removed to Cameron, Milam county, Texas. There was an outbreak of diphtheria at Cameron in December, and Dr. Lott was placed in charge of the epidemic.

---

**Dr. W. F. Starley, Jr.,** of Tyler, Texas, a graduate of the Texas Medical College (1893), has been elected resident student of the John Sealy Hospital, Galveston.

---

**The Samuel D. Gross Prize.**—The Second Quinquennial Prize of one thousand dollars, under the will of the late Samuel D. Gross, M. D., will be awarded January 1, 1900. The conditions annexed by the testator are that the prize "Shall be awarded every five years to the writer of the best original essay, not exceeding one hundred and fifty printed pages, octavo, in length, illustrative of some subject in surgical pathology or surgical practice, founded upon original investigations, the candidates for the prize to be American citizens.

It is expressly stipulated that the successful competitor, who receives the prize, shall publish his essay in book form, and that he shall deposit one copy of the work in the Samuel D. Gross Library of the Philadelphia Academy of Surgery.

The essays, which must be written by a singe author in the English language, should be sent to Dr. J. Ewing Mears, 1429 Walnut St., Philadelphia, before January 1, 1900.

Each essay must be distinguished by a motto, and accompanied by a sealed envelope bearing the same motto, and containing the name and address of the wrtter. No envelope will be opened except that which accompanies the successful essay.

The committee will return the unsuccessful essays if reclaimed by their respective writers, or their agents, within one year.

The committee reserves the right to make no award if the essays submitted are not considered worthy of the prize.

---

**Dr. J. O. Evans,** of Hondo, Mexico, a brother of State Quarantine Inspector A. H. Evans, of Eagle Pass, was married in Austin on December 12th, ult., to Miss Maud Shelley, of this city.

---

**Fourth Annual Report:** Texas State Insane Asylum, San Antonio. The JOURNAL has received the report for the year ending October 31, 1895. Superintendent W. B. Worsham makes a good showing, his first year:

| | | |
|---|---|---|
| Patients on hand Nov. 1, 1894 | | 235 |
| Admitted during year | | 103 |
| | | 338 |
| Discharged restored | 63 | |
| Discharged improved | 20 | |
| Discharged unimproved | 1 | |
| Escaped | 1 | |
| Died | 15— | 100 |
| Remaining on hand | | 238 |

Sixty-three discharged cured (about 20 per cent. of the entire population) is a very extraordinary showing.

Six died of consumption, three of apoplexy, three of status epilepticus, two of general paresis, and one from exhaustion from acute melancholia. Cost of maintenance per capita, $170.84.

---

**Significant.**—A few miles north from Cincinnati a family was prostrate with typhoid, the members of which were positively sure that their affliction was not due to drinking impure water. Their well had not gone dry, and the water was clear and sparkling as ever. The family physician made a bucket of brine and threw it into the privy vault. In a few hours the beautiful clear water in the well was too brackish to drink. Further arguments were unnecessary.—*Lancet-Clinic.*

---

**"Blocks of Three."**—To induce prompt renewals and new subscriptions, the JOURNAL makes this offer for 1896: The subscription price is $2.00 a year. We will send three copies one year for $4.00, all new subscribers, or one renewal and two new names. That is, any one, subscriber or not, who will send us two new subscribers and $4.00, will receive the JOURNAL one year *free* of charge.

---

**The Thirty-eighth Annual Report** of the Trustees and Superintendent of the Texas Institute for the Blind, Austin, Texas, for the year ending November 1, 1895, Dr. E. P. Becton, Superintendent, has been received. It embraces the oculist's report, Dr. H. L. Hilgartner. The report shows the institute in a satisfactory condition. The pupils have many of them been taught industries by which they will be able to support themselves, and will be no longer a burden to the State. Some have had their vision restored; others partly restored. The following from the oculist's report will be interesting:

"With reference to the cases of hopeless blindness found from year to year in the blind institute, I desire to call attention to a matter of very grave importance. Over fifteen per cent. of the cases of total blindness recorded from year to year on the rolls of this school are clearly and demonstrably due to an original disease of infancy, known as 'babies' sore eyes,' which attacks the child when but three days old, and, unless arrested, induces total and irretrievable blindness within a few days. This disease is amenable to treatment in almost every case, and would rarely occur under proper care on the part of the midwives and nurses.

"A legislative enactment requiring such parties promptly to bring these cases to the attention of the physician, was proposed by me in t he lat legislature. The bill was favorably reported by the House committee, but adversely by the committee of the Senate, and was finally left pending for lack of time for its consideration. I deem it a matter of such great importance that I can not refrain from once more alluding to it, and reiterating my conviction of its seriousness and urgency.''

---

**Dr. J. A. Throckmorton,** of Houston, brother of the late ex-Governor Throckmorton, died in Houston December 28th. Age 75.

---

**Dr. John Preston,** ex-Superintendent State Lunatic Asylum, who for some months has been residing at Austin, has removed to Lockhart, where he will succeed to the practice of Dr. Eugene Clark. Dr. Clark will retire for the present from the practice and spend some months in New Orleans at the eye and ear hospitals.

---

**We are** requested by the Fort Worth Pharmacy Company, of Fort Worth, Texas, to say, that they will be pleased, upon application, to mail their Surgical Instrument Catalogue and Price List to any member of the profession.

Their catalogue quotes over six thousand articles and gives about the same number of cuts or figures of the articles.

They will also be pleased, at request, to mail special catalogues of Electric Batteries, Microscopes, Physician's Chairs and Tables and compressed air machines, which are now so successfully used by specialists in the treatment of throat and catarrhal diseases.

---

**Important Meeting.**—The fifth semi-annual meeting of the Central Texas Medical Association will be held at Waco, January 14th and 15th, instant. Dr. J. D. Law, of Salado, is President, and Dr. M. L. Graves, of Waco, in Secretary. The following invitation has been issued:

''Dear Doctor:—Again we have the pleasure of extending you a very urgent invitation to attend the fifth semi-annual meeting of the Central Texas Medical Association, convening in Waco on the second Tuesday in January. It is needless to remind you of the great advantage of active society work; you know that the collision of mind with mind, and of thought with thought, energizes and stimulates the mental digestive power, and makes you a better physician. The State is throbbing with the heartbeats of progress, and not a breeze sweeps our prairies, or a zephyr lingers in our forest, but is attuned by the music of the nineteenth century civilization; all resulting from combined and concentrated thought. Doctor, come, and let us put away from us the satire which scourges and the anger which brands, and be determined to build in Central Texas an Association whose brain will be the temple of wisdom, and whose soul the temple of professional and fraternal honor.

"Yours fraternally,

"JARRETTE D. LAW, M. D., President.

The programme is very attractive, and papers will be read as follows: "Mammitis, Prevention and Treatment."—R. C. Nettles, Marlin. "Scarlet Fever."--H. C. Black, Waco. "Fractures, The Best Line of Treatment."—A. C. Scott, Temple. "Endometritis, Treatment."—O. I. Halbert, Waco. "The Use and Abuse of Calomel."—W. E. Brown, Gatesville. "What Are the Most Reliable Signs and Symptoms of Approaching Insanity?"—D. R. Wallace, Waco. "Castration for Crime as Preventive and Curative Treatment."—J. M. Frazier, Belton. Discussion, A. M. Curtis, Waco, and W. B. Carpenter, Mart. "Causes and Treatment of the Uric Acid Diathesis."—N. A. Olive, Waco. "Acute and Chronic Tonsilitis, Treatment."—J. M. Strayhorn, Bartlett. "The Use and Abuse of Opium and Its Alkaloids."—W. O. Wilkes, Waco. "Diagnosis and Treatment of Intestinal Obstruction."—W. R. Blailock, McGregor. "Causes and Treatment of Ulceration of Bowels."—W. A. Howard, Waco. "Diagnosis and Treatment of Pharyngeal Catarrh."—T. J. Hubbert, Hico. "Management of Normal Labor."—M. D. Knox, Hillsboro. "Iritis."—C. Guy Reily, Waco.

---

**A pretty calendar** for 1896 has been sent us by Messrs. Parke, Davis & Co., for which we return thanks. It is a lithograph representing Thomas O'Cat, Esq., in bed with his head bandaged, evidently suffering from the effects of a spree. By the couch stands a table, on which are Parke, Davis & Co.'s well known remedies—fl. ext. kola nut—amongst others; quite suggestive. The calendar is quite ornamental as well as useful, and is very acceptable. We always like to have a calendar handy, so as to be able, without loss of time, to tell the bill collectors when to call again (the days when we will be "out"). Thanks.

---

**The Growth of Reform in Popular Favor.**—Illustrating forcbly the intolerance of legislatures to suggestions of reform, and especially from physicians, and also showing that a change has taken place in public sentiment with reference to substituting castration for capital punishment, the following incident is related. It is a fact. About twenty-five years ago Dr. Gideon Lincecum, of Washington county, Texas, a very able physician and a world-distinguished scientist; one who had been complimented even by foreign governments with honorary degrees, diplomas and medals, holding that capital punishment is both wrong, and useless as a deterrent of crime, appeared before a Texas legislature and advocated in the ablest manner the substitution of castration of criminals,—pleading in the name of humanity and of science, progress and reform, to so amend the statutes. There was a shout of ridicule that made the State ring for years. The subject became a by-word and a standing joke. The learned doctor was denounced as a crank, and ridiculed on every hand. (He was a little in advance of his age; that is all.) A short time after this occurred, two distinguished politicians of that day, Judge B. and Col. T. H., traveling, had occasion to stop all night at the hospitable home of the venerable doctor. They occupied a bed together up stairs, these two, and away in the night the judge awoke, and found the lamp burning, and his friend, over by the wash stand, doing *something with a towel* between his legs. "Hallo, Colonel, what are you doing?" said the Judge. "Ah, I just got to thinking," said the Colonel, "there's no telling what that old castration crank, Lincecum, down stairs, might take it into his head to do, and I thought I'd better look a little out for mine."

At the last session of the Texas legislature a bill of the kind was introduced and found favor. It had to give way, however, to other matters. Texas will yet lead in this much needed reform.

---

The *College and Clinical Record* will be hereafter known under the name of "*Dunglison's College and Clinical Record:* A Monthly Journal of Practical Medicine."

---

**The Mote and the Beam.**—Shrady, of the *New York Medical Record*, criticises the custom on the part of the "newer journalism" of publishing, under the head of medical news, personal items of doctors, such as often appear in the TEXAS MEDICAL JOURNAL, and condemns it; says it is not news. He instances "Dr. S., of Dallas, injected in his arm three grains of strychnia, by mistake, for cocaine, a drug which he was accustomed to take," and similar items, and says: "We hope and believe that most of the discreditable acts thus reported, relate to men who are not really educated and qualified physicians." There is where Bro. Shrady got left. The doctor referred to in the paragraph above, and in fact most of those mentioned in the "Red-Back" from time to time, are, or were, more or less prominent; recognized as representative men.

All the same, the *Record*, we notice, most frequently copies our personal items, and without credit, however much he pretends to condemn the practice. The funny part of it is, we publish such personal items of doctors under the head of "Medical News and Miscellany." If Dr. Shrady doesn't want to call them "Medical News," let him call them "Miscellany." Funnier still, Shrady calls it "Criminal News." Is there anything criminal in unintentional suicide? Wake up, doctor; journalism is progressive, and you have fallen behind.

---

**Criminology.**—The Chicago Medico-Legal Society will have a meeting January 12, inst., at which will be considered for the most part the subject of crime nnd criminals. Maj. McClaughry, of the State Reformatory at Pontiac, Illinois, will read a paper on the "Habitual Criminal." Dr. Hamilton D. Wey, of the State Reformatory at Elmira, New York, will read a paper on the "Economical Aspects" of the Treatment of Crime and Criminals. Mr. W. S. Elliott, the criminal lawyer of Chicago, a paper on the "Legal Aspects" of the Subject, and Dr. F. E. Daniel, of Austin, Texas, will contribute "A Plea for Reform in Criminal Jurisprudence." It will be a kind of Congress on Crime. Doctors are beginning to believe that crime is a disease and needs medical men to deal with it, and that in time criminals will pass under the care of the medical profession to a large extent, in the same manner that lunatics and inebriates have; they were formerly "punished."

---

**Too Good to Be Lost.**—Some time ago a distinguished and popular physician and an equally popular Presbyterian minister, were both "held up" on the streets of a flourishing Texas city, and robbed. It occurred near the center of the business district, and was just after nightfall; a bold, and, of course, unexpected deed. They were robbed of their watches and money. The robber, a young fellow of twenty-two, was caught, and sent to the penitentiary. Another minister, a special friend of the doctor, at the time absent from the city, heard of it, and, in a spirit of fun, wrote the doctor, asking what time it was by his watch.

The doctor replied to the inquiry at some length. After the lapse of years, the JOURNAL got hold of it, and, considering it too good to be lost (and although it may not be "medical news," and therefore begging the *Record's* pardon), we give it here:

"HOLD UP YOUR HANDS, DOCTOR!"

My Dear Sir:—In language brief
  I'll tell the mournful story,
Of how the highway robber chief
  Involved me in a foray.
He had often heard the preacher say
  That earth was full of booty;
To prey and watch, and watch and prey,
  Fulfilled man's highest duty.
And heard them tell of Joshua's fights,
  When Moses, Hur, and Aaron
"Held up their hands," till 'Malekites
  All patronized old "*Charon*."
Inspired to lead itinerant bands,
  That march right on to glory,
He made men lift their trembling hands,
  To ward off—purgatory.
With lofty *aim*, and nerve of *steel*,
  And "time," *too well* selected,
He made a Presbyterian feel
  That Saints were not elected.
Predestined, in an hour of need,
  To give his gold an' silver,
Was enough to jar an old Scotch creed,
  Or make a round-head quiver.
No Methodist with common sense
  An argument could stand
That *shot* him forward, right or wrong,
  To Canaan's happy land.
I handed over all I had—
  My watch, and chain, and money;—
The exchange made me feel right bad,
  But others thought it funny.
The neophite there made a botch,
  And all men now disdain him;
He keeps about him *still* a "watch,"
  But iron links enchain him.

No starlight lingers in his cell,
No loved ones 'round him sighing,
The clanging bell alone will tell
That hours are slowly dying.

Poor erring boy! may drifting years
Drive o'er his crimes oblivion's wave!
May mother's prayers, and mother's tears,
Yet save him from a felon's grave.
But never, while the planets roll,
Nor while the sun in splendor stands,
Shall I forget the robber bold,
Who gently said—"Hold up your hands."

